# Pulsatilla Saponin B4 Alleviates H_2_O_2_-Induced Oxidative Stress and Apoptosis via the AMPK/Nrf2 Pathway in Bovine Mammary Epithelial Cell Models

**DOI:** 10.3390/antiox15030294

**Published:** 2026-02-27

**Authors:** Hao Zhang, Shouli Yi, Panpan Ding, Baocheng Hao, Dan Shao, Shengyi Wang

**Affiliations:** 1 Key Laboratory of Veterinary Pharmaceutical Development of Ministry of Agriculture, Key Laboratory of New Animal Drug Project, Lanzhou Institute of Husbandry and Pharmaceutical Sciences, Chinese Academy of Agricultural Sciences, Lanzhou 730050, China; 2 College of Veterinary Medicine, Gansu Agricultural University, Lanzhou 730070, China; 3 College of Animal Science and Technology, Guangxi University, Nanning 530004, China; 4 College of Veterinary Medicine, Anhui Agricultural University, Hefei 230036, China

**Keywords:** Pulsatilla saponin B4, BMECs, oxidative damage, apoptosis

## Abstract

The elevated metabolic demands of lactation in dairy cows cause an excess of reactive oxygen species (ROS) in the mammary tissue, which disrupts redox homeostasis and ultimately induces oxidative stress. This oxidative stress directly damages mammary epithelial cells, reduces milk yield and quality, and exacerbates oxidative damage in the mammary gland, ultimately leading to significant economic losses. Therefore, alleviating oxidative stress is essential to safeguard the health of dairy cow mammary glands and ensure farming profitability. Pulsatilla saponin B4 (PSB4), a triterpenoid saponin monomer derived from the roots of *Pulsatilla chinensis*, possesses antioxidant activities. However, its protective effect against oxidative injury in bovine mammary epithelial cells (BMECs) and the exact mechanisms are not fully elucidated. Therefore, this study aims to elucidate the specific protective effects and mechanisms of PSB4 against oxidative damage induced by hydrogen peroxide (H_2_O_2_). The results demonstrated that PSB4 effectively alleviates oxidative stress on two fronts: by enhancing the activities of superoxide dismutase (SOD) and glutathione peroxidase (GSH-Px) to boost total antioxidant capacity (T-AOC), and by significantly reducing malondialdehyde (MDA) levels and suppressing excessive ROS production. Mechanistically, PSB4 primarily functions by enhancing the nuclear relocation of nuclear factor erythroid 2-related factor 2 (Nrf2) and upregulating antioxidant response genes. Furthermore, PSB4 effectively reduced H_2_O_2_-induced apoptosis in BMECs, a finding jointly confirmed by JC-1 assay (effectively reversed mitochondrial depolarization) and flow cytometry (showing reduced apoptotic rates). This protective effect was linked to the normalization of apoptosis-associated protein expression, primarily through an increased B-cell lymphoma 2 (BCL2)/BCL2-associated X Protein (Bax) ratio and decreased cysteinyl aspartate-specific proteinase 3 (Caspase-3) expression. Notably, these protective effects of PSB4 could be antagonized by an AMP-activated protein kinase (AMPK)-specific inhibitor (Compound C, CC). Overall, this preliminary study confirms that at the tested concentrations, PSB4 exerts a protective effect against oxidative damage in BMECs, likely through modulation of the AMPK/Nrf2/Caspase-3 signaling axis. These findings provide a rationale for future in vivo studies and support the potential development of PSB4 as a nutritional supplement or therapeutic agent to alleviate oxidative stress and improve mammary health in dairy cows.

## 1. Introduction

Oxidative stress acts as a critical risk factor in dairy production by directly impairing bovine mammary gland health, compromising raw milk quality, and increasing dependency on conventional antimicrobials along with the associated residue risks. This systematically undermines dairy product safety and ultimately threatens the sustainability of the entire dairy industry. BMECs perform critical functions including milk synthesis, barrier defense, and immune regulation. Therefore, maintaining their health is fundamental to ensuring high-quality milk production and structural stability of the mammary gland [1,2,3]. Under normal circumstances, factors such as high-yield metabolic pressure, infection, inflammation, and stress can result in persistently elevated ROS levels. The synthesis of ROS exceeds the scavenging capacity of mammary tissue (i.e., redox imbalance), which is the direct cause of oxidative stress in mammary tissue [4]. In addition, excessive accumulation of ROS can induce apoptosis in BMECs by activating multiple apoptotic pathways. This large–scale apoptosis leads to the collapse of alveolar structures and damages the blood-milk barrier, ultimately causing the loss of lactational function and breakdown of immune defense, which constitutes a core mechanism of mammary gland injury and functional decline [5,6,7]. Therefore, maintaining stable levels of ROS in BMECs is vital to preserve the structural integrity of the mammary gland, sustain normal lactation function, and regulate immune homeostasis [8,9,10].

As a key nuclear transcription factor, Nrf2 not only regulates cellular antioxidant defenses, but its dysfunction is also implicated in the onset and progression of several diseases [7,11]. Under oxidative stress, Nrf2 acts as the core sensor of oxidative damage [12]. Upon activation, it binds to the ARE and regulates the expression of various downstream redox-regulating genes (including *HO-1*, *GCLC*, *GCLM*, and *NQO1*), thus enhancing cellular antioxidant defense capacity [13]. AMPK serves as a crucial dual sensor for cellular energy and oxidative stress [14]. Under manageable oxidative stress, activated AMPK triggers the Nrf2-mediated “antioxidant program” to eliminate excess ROS, thereby alleviating oxidative damage. However, when oxidative stress becomes uncontrollable, a massive surge of ROS overwhelms the defensive capacity of the Nrf2 pathway. In this situation, AMPK’s role shifts from a protective regulator to an amplifier of apoptotic signals. It phosphorylates and activates pro-apoptotic proteins, elevates outer mitochondrial membrane permeability, and promotes cytochrome C (cytc) release, thereby activating the caspase cascade. This ultimately results in pronounced activation of Caspase-3 and the initiation of apoptosis, leading to aggravated oxidative stress, widespread cellular apoptosis, and tissue injury [15,16,17,18]. Moreover, research has confirmed that the equilibrium between BCL2 and Bax protein levels governs the onset of apoptosis, while Caspase-3 is responsible for executing the terminal phase of programmed cell death [19,20,21]. Consequently, in studies on mammary oxidative damage, the BCL2/Bax ratio and Caspase-3 activity not only serve as crucial biomarkers for evaluating the extent of apoptosis, but also as key targets for mammary gland protection and anti-apoptotic interventions.

**Figure 1:**
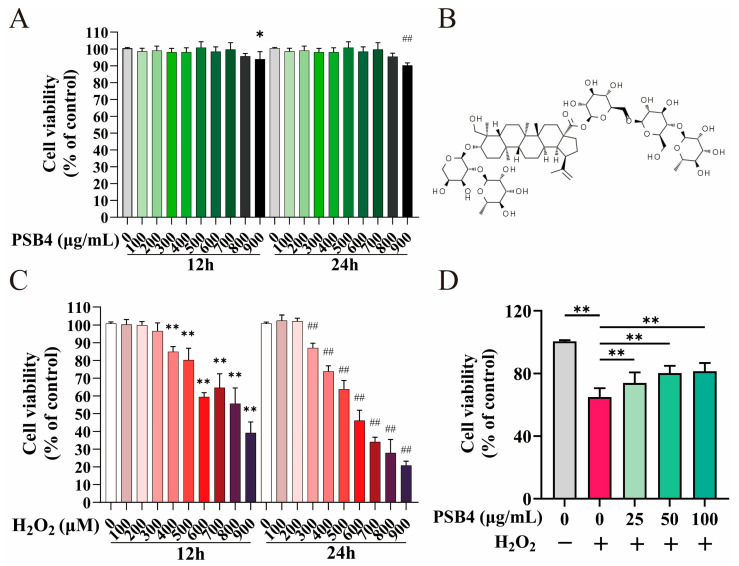
The impact of PSB4, H_2_O_2_, and their combination (PSB4 + H_2_O_2_) on BMEC viability. (**A**) BMECs were incubated with varying doses of PSB4 (0–900 μg/mL) for different durations (12 h or 24 h), and cell survival was quantified (*n* = 9). (**B**) Chemical structure of PSB4. (**C**) BMECs were treated with varying concentrations of H_2_O_2_ (0–900 μg/mL) for different durations (12 h or 24 h), and cell viability was assessed (*n* = 9). (**D**) Evaluation of the cytoprotective effect of multiple concentrations of PSB4 (25, 50, or 100 μg/mL) against H_2_O_2_-induced injury in BMECs (*n* = 9). Data are presented as mean ± standard error. * *p* < 0.05 and ** *p* < 0.01, as well as ^##^ *p* < 0.01, indicate significant differences compared with the corresponding blank group.

Many natural extracts are valuable resources for antioxidant active ingredients [22,23]. PSB4 is a triterpenoid saponin monomer extracted from the roots of *Pulsatilla chinensis* with the molecular formula C_59_H_96_O_26_ (Figure 1B). Emerging evidence indicates that PSB4 demonstrates diverse pharmacological capacity, encompassing anti-inflammatory, antioxidative, antibacterial, and antitumor effects [24,25,26,27,28]. Notably, PSB4 has been clinically used in the treatment of ulcerative colitis, where it exerts significant protective effects on intestinal epithelial cells by alleviating oxidative stress, suppressing inflammatory responses, and preserving epithelial structural integrity [29,30]. Interestingly, bovine mastitis is characterized by mammary epithelial cell injury, oxidative stress, and inflammatory damage, pathological features that closely resemble intestinal epithelial damage in colitis. This suggests that PSB4 may similarly exert protective effects on injured mammary epithelial cells. Furthermore, PSB4 possesses favorable pharmacological properties, including high structural stability, low cytotoxicity, and proven efficacy in inflammatory and oxidative stress-related disorders [24,25]. Importantly, our research group recently reported that PSB4 effectively alleviates clinical mastitis in lactating dairy cows [31], highlighting its translational potential in bovine health. Collectively, these lines of evidence establish a foundation for the specific selection of PSB4 among various triterpenoid saponins in this study. However, the underlying mechanisms by which PSB4 protects BMECs from oxidative injury remain unclear. Given the central role of oxidative stress in the pathogenesis of mammary gland dysfunction, elucidating the antioxidant and anti-apoptotic mechanisms of PSB4 in BMECs may provide a scientific basis for its application as a potential therapeutic or nutritional intervention to improve mammary health and milk quality in dairy cows. Accordingly, the objective of this study is to evaluate the protective effects of PSB4 at the tested concentrations against oxidative stress and apoptosis in BMECs and to further investigate the signaling pathway mechanisms underlying its action.

## 2. Materials and Methods

### 2.1. Materials

PSB4 (purity > 98%; CAS 129741-57-7) was obtained Solarbio (Beijing, China) and dis solved in RPMI 1640 medium. H_2_O_2_ (30% *w*/*w* solution) was obtained from Solarbio (Beijing, China). Compound C (CC, 866405-64-3, Shanghai, China) was obtained from MCE.

### 2.2. Cell Cultivation and Experimental Processing

The BMECs used in this study are a commercially available low-passage (Passage 4) bovine mammary epithelial cell line purchased from Jennio Biotechnology Co., Ltd. (Guangzhou, China). The culture conditions were RPMI 1640 (Gibco, Shanghai, China) + 10% FBS, 37 °C, 5% CO_2_. Digestion was performed with 0.25% trypsin-EDTA and quenched with complete medium. After centrifugation, the cells were resuspended and passaged.

Standard treatment group: BMECs were stimulated with 500 μM H_2_O_2_ for 12 h, followed by treatment with 25, 50, and 100 μg/mL PSB4 for another 12 h.

AMPK inhibition group: BMECs were stimulated with 500 μM H_2_O_2_ for 12 h, followed by co-incubation with 100 μg/mL PSB4 and 10 μM CC (MCE, Shanghai, China) for 12 h. The dosage and administration of CC were based on previous validated studies in BMECs [32,33].

### 2.3. Cytotoxicity Detection

BMECs viability was assessed using the CCK-8 (NCM Biotech, Suzhou, China), performed as specified by the manufacturer.

### 2.4. Antioxidant Capacity Measurement

After processing BMECs according to the experimental protocol, the corresponding samples were collected. The levels of key antioxidant indices (T-AOC, A015-3-1; SOD, A001-3-1; GSH-Px A005-1-2, Nanjing, China) and the oxidative damage product MDA (A003-4-1, Nanjing, China) in BMECs were measured using the corresponding assay kits from NJJCB.

### 2.5. Monitoring of ROS

BMECs were treated under different conditions, and intracellular ROS levels were measured using the DCFH-DA fluorescent probe (Beyotime, Shanghai, China) according to the method described by Shao D et al. [34]. Fluorescence images were captured with a Zeiss LSM700 confocal microscope (ZEISS, Oberkochen, Germany), and fluorescence intensity was quantified using ImageJ software (Version 1.53e, NIH, Bethesda, MD, USA).

### 2.6. ΔΨm Assay

Mitochondrial membrane potential was detected by flow cytometry using a JC-1 fluorescent probe kit (Beyotime, Shanghai, China), and changes in membrane potential were evaluated based on the ratio of JC-1 aggregates to monomers.

### 2.7. Immunofluorescence Staining

Immunofluorescence was performed to evaluate the nuclear translocation of Nrf2 in BMECs. Following the treatment protocol established by Dong et al. [35], cells were fixed and immunostained with an anti-Nrf2 primary antibody (1:500, ab137550, Abcam, Cambridge, MA, USA) and subsequently with an Alexa Fluor 594-labeled goat anti-rabbit secondary antibody (1:500; Thermo Fisher, Waltham, MA, USA). Nuclei were visualized with DAPI. Images were acquired using a Nikon A1Rsi confocal laser scanning microscope (Nikon Corporation, Tokyo, Japan) with appropriate filters for DAPI (nuclei) and Alexa Fluor 594 (Nrf2).

### 2.8. Real-Time PCR

Total RNA was isolated with TRNzol Universal reagent (TIANGEN, Beijing, China; Cat. DP424), a commercial reagent based on the classic TRIzol (phenol-guanidine isothiocyanate) method. RNA purity and concentration were measured with an Eppendorf BioPhotometer^®^ D30 (Eppendorf, Hamburg, Germany). The subsequent steps for gDNA elimination and cDNA synthesis were performed according to the manufacturer’s instructions (Biotechnology, Changsha, China). qPCR was conducted on an Applied Biosystems system with SYBR Green Pro Taq HS premixed reagent. Gene expression levels were normalized to β-actin and calculated using the 2^−ΔΔCt^ method. All primers used (Table 1) were synthesized by Xi’an Tsingke Biotechnology Co., Ltd. (Xi’an, China).

### 2.9. Protein Extraction and Immunoblotting Analysis

Total protein from BMECs in each treatment group was extracted using RIPA lysis buffer. Protein concentration was determined by the BCA method, and samples were aliquoted at a concentration of 30 μg/10 μL. The specific procedure for Western blotting was performed according to the method described by Shao D et al. [34]. The reagents used are listed as follows: BCL2 (26593-1-AP,1:1000, Proteintech, Wuhan, China), Cleaved Caspase-3 (25128-1-AP,1:1000, Proteintech, Wuhan, China), Bax (50599-2-IG, 1:1000, Proteintech, Wuhan, China), Nrf2 (12721T, 1:2000, CST, Danvers, MA, USA), AMPK (2532S,1:2000, CST, Danvers, MA, USA), p-AMPK (2531S,1:2000, CST, Danvers, MA, USA), β-actin (4967S,1:5000, CST, Danvers, MA, USA); IgG-HRP (1:5000; Proteintech, Wuhan, China); high-sensitivity enhanced chemiluminescence (ECL) reagent (NCM Biotech, Suzhou, China).

### 2.10. Assessment of Cellular Apoptotic Activity

Apoptosis rates of BMECs in each treatment group were detected using an Annexin V-FITC staining kit (Beyotime, Shanghai, China). Subsequently, signal acquisition and data processing were performed with a BD FACSAria III flow cytometer (BD Biosciences, San Jose, CA, USA) to analyze apoptosis rates of BMECs.

### 2.11. Statistical Analysis of Experimental Data

The Shapiro–Wilk test was used to evaluate the normality of all data, and the Levene test was used to analyze the homogeneity of variance. All comparisons included independent biological replicates. Data were expressed as mean ± standard error (mean ± SEM). Statistical analysis and chart drawing were performed using GraphPad Prism 9 software (GraphPad, Boston, MA, USA). Differences between groups were evaluated by one-way analysis of variance combined with Dunnett’s post hoc test. Statistical significance criteria were set as * *p* < 0.05 and ** *p* < 0.01.

## 3. Results

### 3.1. PSB4 Ameliorates H_2_O_2_-Induced Reduction in BMEC Viability

This investigation was designed to identify the cytotoxic range of PSB4 in BMECs and to optimize H_2_O_2_ concentration and exposure time for establishing an oxidative stress model. As shown in Figure 1A, BMECs viability showed no significant alterations following treatment with 100–800 μg/mL PSB4 for 12 h or 24 h as opposed to the unstimulated control (*p* > 0.05); by contrast, 900 μg/mL PSB4 markedly impaired BMEC viability at both sampling times (*p* < 0.05). Thus concentrations below 900 μg/mL were defined as non-cytotoxic. Notably, in subsequent tests, concentrations exceeding 100 μg/mL did not exhibit enhanced protective effects. The effects of 0–900 μM H_2_O_2_ on BMEC viability after 12 h or 24 h of exposure are illustrated in Figure 1C. Challenge with H_2_O_2_ (400–900 μM) for 12 h significantly reduced cell viability (*p* < 0.01), and exposure to 300–900 μM for 24 h also caused a significant decrease (*p* < 0.01). Based on these results, 500 μM H_2_O_2_ with 12 h stimulation was employed as the oxidative stress optimal condition for subsequent investigation, and 25, 50, and 100 μg/mL were selected as the therapeutic concentrations of PSB4. Furthermore, as illustrated in Figure 1D, relative to the untreated control group, H_2_O_2_ (500 μM) stimulation significantly lowered BMECs viability (*p* < 0.01). Relative to the H_2_O_2_ group, different concentrations of PSB4 (25, 50 and 100 μg/mL) observably restored cell survival (*p* < 0.01). These findings confirmed that PSB4 potently attenuated the detrimental effects of H_2_O_2_ on BMEC viability.

### 3.2. PSB4 Alleviates Oxidative Stress in H_2_O_2_-Induced BMECs

**Figure 2:**
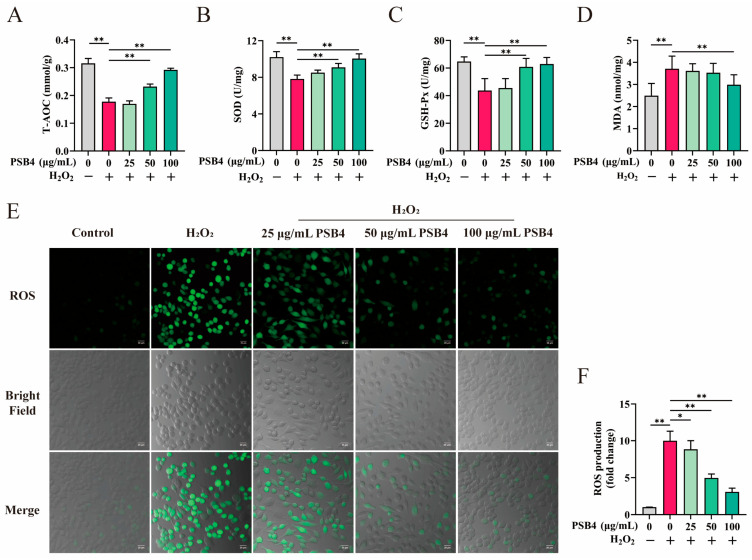
Effects of PSB4 on T-AOC, SOD, GSH-Px, MDA, and ROS levels in H_2_O_2_-induced BMECs. BMECs were exposed to H_2_O_2_ (500 μM) for 12 h, followed by incubation with PSB4 (25, 50, or 100 μg/mL) for a further 12 h. (**A**–**D**) The activities of T-AOC, SOD, and GSH-Px, along with MDA content, were measured in BMECs (*n* = 3) (**E**) Representative confocal images of ROS staining in BMECs. Green: DCFH-DA fluorescent probe (ROS); Gray: bright-field (cell morphology); Merged: overlay of green fluorescence and bright-field. Scale bar = 20 μm. (**F**) Statistical comparison of relative fluorescence intensity among treatment groups (*n* = 3). Data are presented as the mean ± standard error of the mean (SEM). Statistical significance is indicated in the figures as * *p* < 0.05 and ** *p* < 0.01.

To elucidate the action of PSB4 on H_2_O_2_-induced oxidative stress, we assessed indicators of antioxidant capacity along with levels of ROS. Figure 2A–D show that H_2_O_2_ brought about a pronounced decrease in T-AOC, SOD, and GSH-Px activity or content (*p* < 0.01), while markedly increasing MDA and ROS levels (*p* < 0.01). In contrast, treatment with PSB4 significantly counteracted the H_2_O_2_-induced reduction in T-AOC, SOD, and GSH-Px (*p* < 0.01), while 100 μg/mL PSB4 also notably suppressed the H_2_O_2_-induced increase in MDA (*p* < 0.01). Furthermore, as depicted in Figure 2E,F, H_2_O_2_ exposure led to pronounced intracellular ROS accumulation. Administration of PSB4 significantly and dose-dependently lowered H_2_O_2_-induced ROS levels (*p* < 0.01). This evidence together suggests that PSB4 mitigates H_2_O_2_-induced oxidative damage in BMECs by restoring antioxidant enzyme activities and attenuating elevations in MDA and ROS levels.

### 3.3. PSB4 Exerted Anti-Apoptotic Effect in H_2_O_2_-Induced BMECs

**Figure 3:**
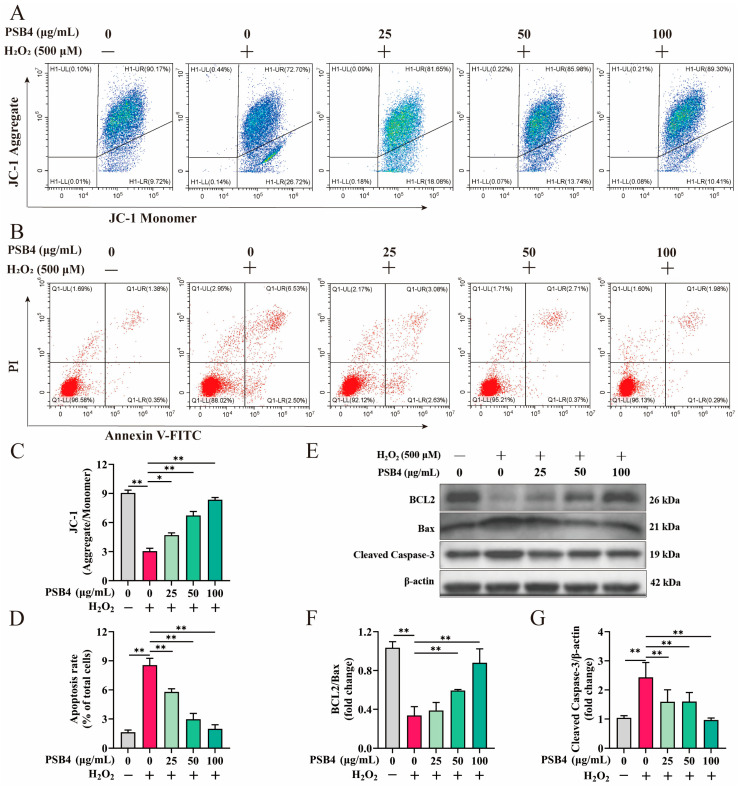
Treatment with PSB4 mitigated H_2_O_2_-induced apoptosis in BMECs. BMECs were first stimulated with H_2_O_2_ (500 μM) for 12 h, after which they were treated with PSB4 (25, 50, and 100 μg/mL) for another 12 h. (**A**) Representative flow cytometry plots from JC-1 staining. Red represents JC-1 aggregates (high ΔΨm), and green represents JC-1 monomers (low ΔΨm). (**B**) Apoptosis in BMECs by flow cytometry. Quadrants: UL (necrotic), UR (late apoptotic), LL (viable), LR (early apoptotic). (**C**) JC-1 (aggregate/monomer) fluorescence ratio in treated BMECs (*n* = 3). (**D**) Statistical analysis of apoptotic cell percentages based on panel B (*n* = 3). (**E**) Analysis of apoptotic proteins (BCL2, Bax, Cleaved Caspase-3) in BMECs by Western blot. The predicted molecular weights for BCL2, Bax, Cleaved Caspase-3, and β-actin were 26 kDa, 21 kDa, 19 kDa, and 42 kDa, respectively. (**F**,**G**) Assessment of relative protein band density for the above targets, with normalization to β-actin; band quantification from Western blots (*n* = 3). Values are expressed as the mean ± SEM. Statistical significance is indicated as follows: * *p* < 0.05, ** *p* < 0.01.

To further investigate the ameliorative effect of PSB4 in response to H_2_O_2_-induced BMEC injury, we examined apoptosis-related indicators. Results showed that H_2_O_2_ treatment significantly reduced the fluorescence ratio of JC-1 aggregates to monomers (*p* < 0.01), while PSB4 intervention reversed this decline in a dose-dependent manner (Figure 3A,C, *p* < 0.01). The detection of apoptosis rate is shown in Figure 3B,D. H_2_O_2_ treatment markedly increased the proportion of apoptotic cells (*p* < 0.01), whereas PSB4 intervention reduced the apoptotic rate in a concentration-dependent fashion (*p* < 0.01). As shown in Figure 3E–G, H_2_O_2_ treatment significantly suppressed the expression of the anti-apoptotic protein BCL2 and reduced the BCL2/Bax protein ratio (*p* < 0.01). In contrast, high-dose PSB4 markedly restored BCL2 expression and increased the BCL2/Bax ratio (*p* < 0.01). Concurrently, Cleaved Caspase-3 protein levels were significantly elevated in the H_2_O_2_ group (*p* < 0.01), and this upregulation was suppressed by PSB4 in a dose-dependent manner (*p* < 0.01). These findings demonstrate the efficacy of PSB4 in mitigating H_2_O_2_-induced damage in BMECs.

### 3.4. PSB4 Modulates AMPK/Nrf2 Signaling in H_2_O_2_-Stimulated BMECs

**Figure 4:**
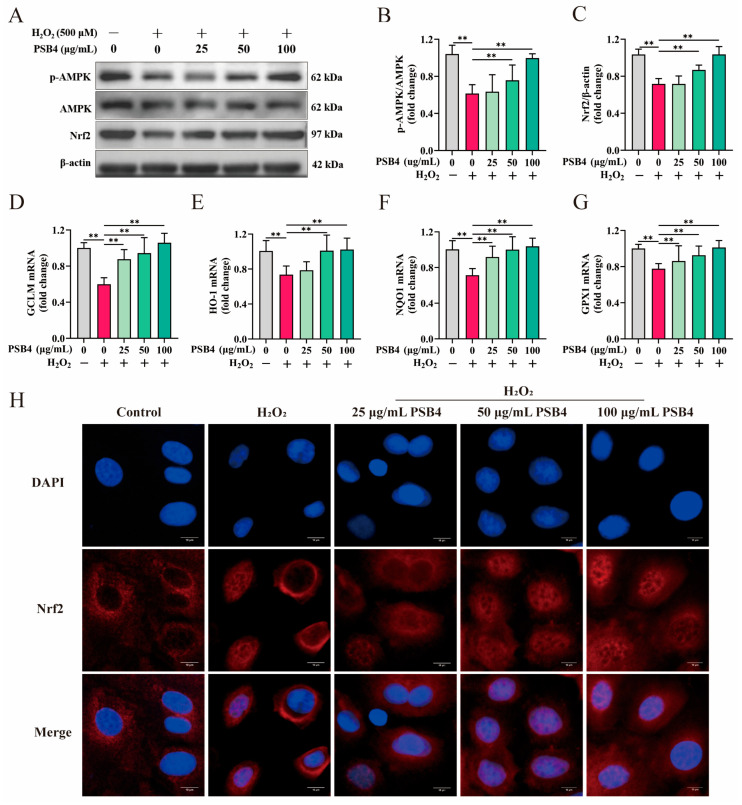
The regulatory effect of PSB4 on the AMPK/Nrf2 signaling pathway. BMECs were first stimulated with H_2_O_2_ (500 μM) for 12 h, followed by treatment with PSB4 (25, 50, and 100 μg/mL) for another 12 h. (**A**) Protein expression of Nrf2, p-AMPK, AMPK, and β-actin in BMECs was analyzed using Western blot. The predicted molecular weights for Nrf2, AMPK, p-AMPK, and β-actin were 97 kDa, 62 kDa, 62 kDa, and 42 kDa, respectively. (**B**,**C**) Quantification of relative band intensity for the respective proteins, normalized to β-actin. Quantification of the bands in the Western blot (*n* = 3). (**D**–**G**) Determination of *GCLM*, *HO-1*, *NQO1* and *GPX1* gene expression via qRT-PCR (*n* = 3). (**H**) Representative immunofluorescence images of Nrf2 nuclear translocation in BMECs under different treatment conditions. Red: Nrf2 (Alexa Fluor 594); blue: DAPI (nuclei). Scale bar = 10 µm; *n* = 3. Values are expressed as the mean ± SEM. Statistical significance is indicated as follows: ** *p* < 0.01.

To investigate the mechanism underlying PSB4’s alleviation of H_2_O_2_-induced oxidative stress in BMECs, we analyzed the expression of key proteins in the AMPK/Nrf2 pathway and their downstream target genes, as well as the nuclear translocation of Nrf2, across different treatment groups. As presented in Figure 4A–C, H_2_O_2_ stimulation significantly inhibited Nrf2 protein expression (*p* < 0.01) and reduced AMPK phosphorylation levels (*p* < 0.01). Treatment with PSB4 dose-dependently restored the suppressive effects of H_2_O_2_ on p-AMPK and Nrf2 expression (*p* < 0.01). Furthermore, PSB4 treatment dose-dependently promoted Nrf2 nuclear translocation as shown by immunofluorescence analysis (Figure 4H). Meanwhile, as shown in Figure 4D–G, PSB4 dose-dependently reversed the downregulation of antioxidant genes (*GCLM*, *HO-1*, *NQO1*, and *GPX1*) induced by H_2_O_2_ stimulation (*p* < 0.01). These findings indicate that PSB4 alleviates H_2_O_2_-induced redox imbalance in BMECs, likely via activation of the AMPK/Nrf2 pathway.

### 3.5. PSB4 Displayed Antioxidative Properties Through AMPK Signaling in H_2_O_2_-Stimulated BMECs

**Figure 5:**
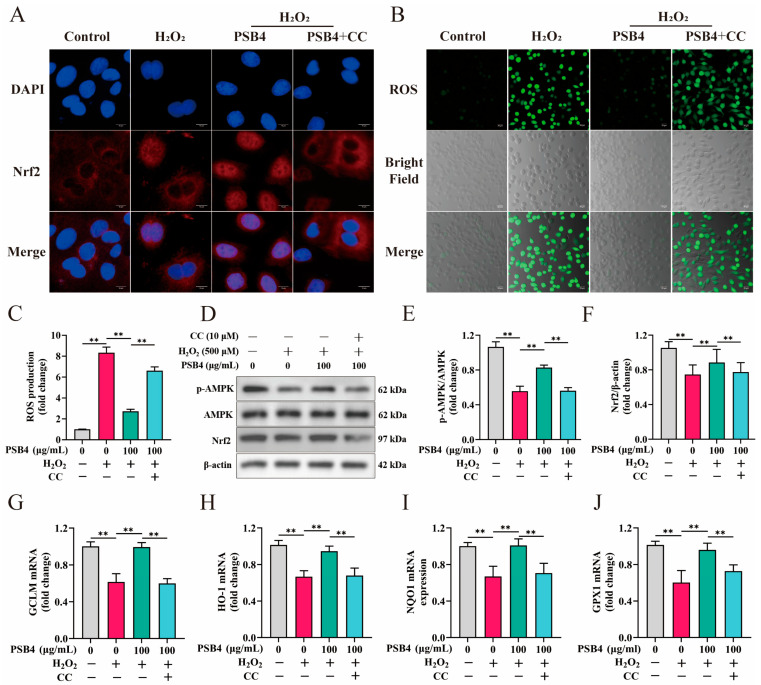
PSB4 demonstrated antioxidative properties by means of AMPK signaling in H_2_O_2_-treated BMECs. BMECs were first stimulated after exposure to H_2_O_2_ (500 μM) for 12 h, followed by a 12 h incubation in combination with PSB4 (100 μg/mL) and CC (10 μM) in the culture medium. (**A**) Representative immunofluorescence images of Nrf2 nuclear translocation in BMECs under different treatment conditions. Red: Nrf2 (Alexa Fluor 594); blue: DAPI (nuclei). Scale bar = 10 µm; *n* = 3. (**B**) Representative confocal images of ROS staining in BMECs. Green: DCFH-DA fluorescent probe (ROS); Gray: bright-field (cell morphology); Merged: overlay of green fluorescence and bright-field. Scale bar = 20 μm. (**C**) Quantitative analysis of relative fluorescence intensity in BMECs across treatment groups (*n* = 3). (**D**) Western blot assay of Nrf2, p-AMPK and AMPK in BMECs. The predicted molecular weights for Nrf2, AMPK, p-AMPK, and β-actin were 97 kDa, 62 kDa, 62 kDa, and 42 kDa, respectively. (**E**,**F**) Evaluation of the relative intensity of the respective protein bands, standardized to β-actin; quantification of the bands from the Western blot (*n* = 3). (**G**–**J**) Gene expression changes in *GCLM*, *HO-1*, *NQO1* and *GPX1* were analyzed using qRT-PCR (*n* = 3). Values are expressed as the mean ± SEM. Statistical significance is indicated as follows: ** *p* < 0.01.

To further investigate if PSB4 mitigates H_2_O_2_-triggered oxidative damage in BMECs through AMPK signaling, we introduced the AMPK-specific inhibitor CC into the experimental system. As presented in Figure 5A, immunofluorescence analysis indicated that PSB4 administration facilitated Nrf2 nuclear translocation, while the addition of CC reversed this effect. Furthermore, H_2_O_2_ exposure resulted in a significant accumulation of intracellular ROS, whereas PSB4 effectively attenuated such ROS accumulation. However, the addition of CC markedly impaired the protective effect of PSB4 (Figure 5B,C, *p* < 0.01). At the protein level, PSB4 treatment significantly counteracted the H_2_O_2_-mediated suppression of Nrf2 protein expression and AMPK phosphorylation (*p* < 0.01), while CC markedly blocked the effect of PSB4 (Figure 5D–F, *p* < 0.01). At the transcriptional level, H_2_O_2_ treatment significantly suppressed the antioxidant response (*GCLM*, *HO-1*, *NQO1*, and *GPX1*), PSB4 intervention markedly increased the expression of these genes, whereas the addition of the AMPK inhibitor CC reversed this effect of PSB4 (Figure 5G–J, *p* < 0.01). Collectively, these results indicate that PSB4 alleviates H_2_O_2_-induced oxidative stress in BMECs by modulating AMPK signaling to activate the downstream Nrf2 pathway.

### 3.6. PSB4 Alleviates H_2_O_2_-Induced Apoptosis in BMECs via the AMPK Pathway

**Figure 6:**
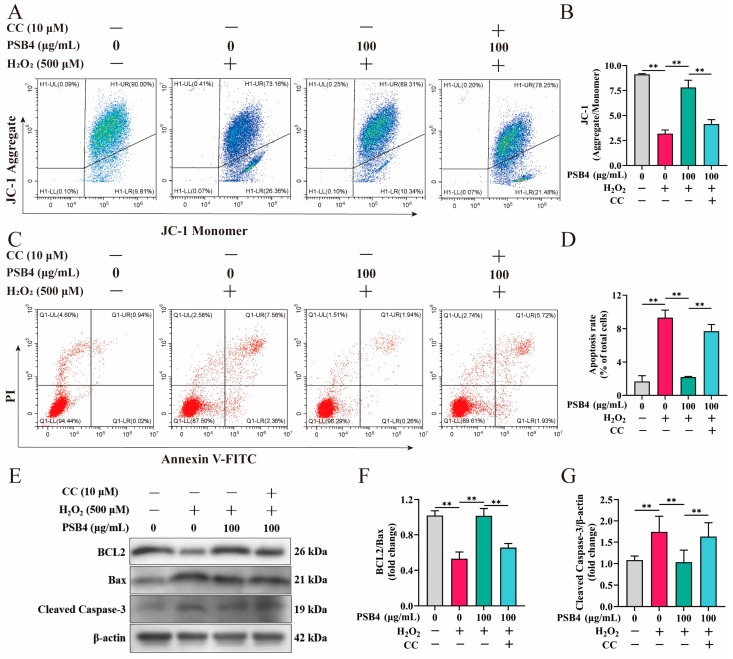
PSB4 regulates apoptosis of BMECs through AMPK signaling. H_2_O_2_ (500 μM) was applied to BMECs for a 12 h treatment period, followed by the addition of PSB4 (100 μg/mL) and CC (10 μM) to the medium for an additional 12 h. (**A**) Representative flow cytometry plots from JC-1 staining. Red represents JC-1 aggregates (high ΔΨm), and green represents JC-1 monomers (low ΔΨm). (**B**) Statistical analysis of fluorescence ratio of JC-1 aggregates versus monomers (*n* = 3). (**C**) Apoptosis in BMECs by flow cytometry. Quadrants: UL (necrotic), UR (late apoptotic), LL (via-ble), LR (early apoptotic). (**D**) Quantitative statistical analysis of apoptotic cell ratio (*n* = 3). (**E**) Western blot analysis of BCL2, Bax, and Cleaved caspase-3 expression in BMECs across treatment groups. The predicted molecular weights for BCL2, Bax, Cleaved Caspase-3, and β-actin were 26 kDa, 21 kDa, 19 kDa, and 42 kDa, respectively. (**F**,**G**) Relative quantification of the respective proteins, normalized to β-actin (*n* = 3). Values are expressed as the mean ± SEM. Statistical significance is indicated as follows: ** *p* < 0.01.

To verify whether PSB4 alleviates H_2_O_2_-induced apoptosis in BMECs via AMPK signaling, we assessed the impact of PSB4 on apoptosis under conditions with and without AMPK inhibition. As shown in Figure 6A,B, the JC-1 staining assay indicated that H_2_O_2_ markedly reduced mitochondrial membrane potential, as evidenced by a decreased aggregate/monomer fluorescence ratio (*p* < 0.01), while PSB4 treatment significantly restored this ratio (*p* < 0.01). Notably, the introduction of CC counteracted the protective influence of PSB4 on mitochondrial membrane potential (*p* < 0.01). Furthermore, flow cytometric analysis revealed that H_2_O_2_ markedly increased the apoptosis rate of BMECs (*p* < 0.01, Figure 6C,D), while PSB4 significantly attenuated H_2_O_2_-induced apoptosis (*p* < 0.01), an effect that was notably reversed by CC (*p* < 0.01). Meanwhile, H_2_O_2_ caused a marked reduction in anti-apoptotic BCL2 expression and a notable rise in Cleaved Caspase-3 levels relative to the control (Figure 6E–G, *p* < 0.01). PSB4 application effectively reversed the H_2_O_2_-mediated suppression of BCL2 and elevation in Cleaved Caspase-3 (*p* < 0.01); however, these effects were substantially blocked by the AMPK inhibitor CC (*p* < 0.01). These results further suggest that PSB4 alleviates H_2_O_2_-induced apoptosis in BMECs via AMPK signaling, thereby mitigating oxidative damage.

## 4. Discussion

Mammary gland tissue represents one of the most metabolically active functional organs in lactating dairy cows. During lactation, robust milk synthesis and secretion significantly boost the aerobic metabolic capacity of mammary tissue, thereby increasing the risk of excessive free radical accumulation. BMECs constitute the primary cellular component of the mammary gland. Excessive accumulation of free radicals impairs the antioxidant capacity and immune function of BMECs, leading to oxidative damage and even apoptosis, which ultimately disrupts normal mammary lactation function and results in decreased milk yield and quality [36]. Therefore, enhancing the antioxidant capacity of BMECs in dairy cows may be an effective strategy to counteract oxidative stress. Emerging studies indicate that the traditional Chinese medicine monomer PSB4 demonstrates anti-inflammatory, antioxidant, antibacterial, and antitumor properties [25,26,27,28,29]. However, research on its application in oxidative stress-related mammary gland diseases remains limited. Accordingly, the current research evaluated the impact of PSB4 on oxidative injury in BMECs triggered by H_2_O_2_ and its associated molecular pathways. This study confirms that PSB4 effectively rescues H_2_O_2_-triggered oxidative stress and apoptosis in BMECs by modulating AMPK signaling to activate the Nrf2 pathway and inhibit the Caspase-3 cascade. These findings provide potential targets for preventing and treating oxidative stress-related mammary disorders in dairy cows and lay a theoretical foundation for developing highly effective natural antioxidants.

During cellular oxidative stress, excessive accumulation of ROS triggers lipid peroxidation, leading to the generation of MDA. This process further exacerbates oxidative stress. Meanwhile, ROS also depletes intracellular antioxidants and inhibits key antioxidant enzyme function, like SOD and GSH-Px. This leads to diminished T-AOC and eventually compromises the cellular antioxidant defense system [37,38]. Consequently, enhancing cellular antioxidant defenses and suppressing excessive ROS production are essential strategies for alleviating oxidative stress. In this investigation, we observed that PSB4 at the tested concentrations significantly inhibited H_2_O_2_-triggered ROS and MDA accumulation in BMECs while simultaneously enhancing the activity of cellular antioxidant enzymes. These results suggest that PSB4 likely exerts its antioxidant effects by both boosting intrinsic antioxidant capacity and inhibiting the overproduction of ROS.

Research indicates that the disequilibrium between elevated ROS production and the endogenous antioxidative defense mechanism serves as an initial trigger for cellular injury, which may subsequently contribute to mammary dysfunction in dairy cows [39,40]. Moreover, the substantial ROS generated during oxidative stress disrupts mitochondrial transmembrane potential, leading to mitochondrial impairment [41,42]. In line with this, our JC-1 staining results demonstrated that H_2_O_2_ stimulation markedly decreased the mitochondrial membrane potential in BMECs, while PSB4 treatment effectively restored it. Damaged mitochondria release additional ROS, which further aggravates apoptosis [43]. The balance between the expression of BCL2 and Bax on the mitochondrial membrane serves as a key factor in regulating mitochondrial stability and cellular apoptosis [44]. BCL2 antagonizes Bax to preserve mitochondrial membrane integrity, thereby suppressing the activation of Caspase-3 and exerting an anti-apoptotic effect [45,46]. Therefore, the BCL2/Bax ratio serves as a pivotal indicator of mitochondrial integrity and cellular commitment to apoptosis, making it a relevant marker for assessing the regulatory effects of interventions on the mitochondrial apoptosis pathway in BMECs. Previous research has indicated that ROS, as key mediators of oxidative stress, can induce the intrinsic apoptotic pathway, resulting in an elevated BCL2/Bax ratio and subsequent activation of Caspase-3 [47]. In summary, oxidative stress is closely associated with the apoptotic process. Therefore, to evaluate the effect of PSB4 on H_2_O_2_-triggered apoptosis in BMECs, we assessed apoptosis-related indicators. The results revealed that PSB4 effectively restored mitochondrial membrane potential and normalized the levels of apoptosis-related proteins, thereby significantly alleviating H_2_O_2_-induced apoptosis.

Upon oxidative stress, nuclear-translocated Nrf2 binds to the antioxidant response element to upregulate transcription of cytoprotective proteins and antioxidant enzymes. This signaling cascade enhances the expression of antioxidant-related genes like *NQO1*, *GPX1*, *HO-1* and *GCLM*, thereby systemically enhancing the cellular oxidative defense capacity. In this study, immunofluorescence staining confirmed that PSB4 treatment effectively promoted the nuclear translocation of Nrf2 in BMECs induced by H_2_O_2_. Moreover, activation of Nrf2 has been shown to suppress the overproduction of ROS and MDA [37,48]. AMPK, a cellular energy sensor [49,50], can promote the nuclear translocation of Nrf2 via phosphorylation, consequently boosting the activity of antioxidative enzymes to scavenge ROS and preserve redox equilibrium [51]. In agreement with this mechanism, our findings revealed that PSB4 markedly activated the AMPK/Nrf2 signaling pathway, indicated by elevated AMPK phosphorylation and augmented Nrf2 expression and nuclear translocation, which collectively contributed to the upregulation of its downstream antioxidant genes. Meanwhile, excessive ROS accumulation can trigger the apoptotic pathway and induce cell death. Therefore, AMPK-driven activation of Nrf2 constitutes a pivotal mechanism to alleviate oxidative damage and inhibit apoptosis through diminishing ROS accumulation [14,52].

In the present research, we observed that PSB4 substantially suppressed the overproduction of ROS triggered by H_2_O_2_ in BMECs and effectively counteracted the H_2_O_2_-triggered inhibition of AMPK phosphorylation and Nrf2 activation, as well as the downregulation of downstream genes of Nrf2. These results suggest that, at the concentrations tested, PSB4 may exert antioxidative and anti-apoptotic effects by modulating AMPK signaling to regulate the Nrf2 pathway, thereby suppressing ROS burst. Although our findings confirm that PSB4 modulates the AMPK/Nrf2 signaling cascade, the precise mechanism by which PSB4 activates AMPK remains to be elucidated. It is unclear whether PSB4 acts as a direct AMPK activator or functions through upstream kinases such as LKB1, a well-established regulator that phosphorylates AMPK to initiate its activation. This uncertainty warrants further investigation. Regarding the anti-apoptotic effects of PSB4, our results demonstrated that H_2_O_2_ treatment reduced the BCL2/Bax ratio and increased the expression of Cleaved Caspase-3 in BMECs, while PSB4 administration effectively reversed these aberrant changes, suggesting that PSB4 exerts its protective effects by inhibiting the intrinsic apoptotic pathway. However, a more comprehensive understanding of its anti-apoptotic mechanism could be achieved by exploring whether PSB4 also regulates the extrinsic apoptotic pathway, such as the Fas/FasL pathway. Given that Cleaved caspase-3 serves as a key executor in both intrinsic and extrinsic apoptotic pathways, PSB4 may potentiate its anti-apoptotic effects through the coordinated modulation of both pathways. This hypothesis warrants further validation in subsequent studies by examining the expression of Fas, FasL and Cleaved caspase-8. To further verify the above conclusion, we employed an AMPK inhibitor for intervention. The results showed that interference with AMPK activity significantly attenuated the protective effect of PSB4 against H_2_O_2_-triggered oxidative damage, while also markedly reducing the anti-apoptotic efficacy of PSB4. While these mechanistic findings are derived from the BMEC model, a recognized platform for initial pathway discovery, their translational relevance is strongly supported by independent clinical evidence. Our prior study demonstrated that PSB4 treatment significantly ameliorated mastitis in lactating milking cows [31], underscoring the physiological significance of the protective mechanisms identified here. Based on these findings, we conclude that PSB4 activates the AMPK/Nrf2 signaling cascade to induce the antioxidative response, thereby eliminating excess ROS in BMECs and mitigating H_2_O_2_-induced oxidative injury. However, the precise mechanism by which PSB4 activates AMPK remains to be elucidated, and whether it also modulates the extrinsic apoptotic pathway warrants further investigation. Future studies examining upstream AMPK kinases such as LKB1, as well as extrinsic pathway markers including Fas, FasL and Cleaved caspase-8, will provide a more comprehensive understanding of the molecular mechanisms underlying PSB4’s cytoprotective effects.

## 5. Conclusions

**Figure 7:**
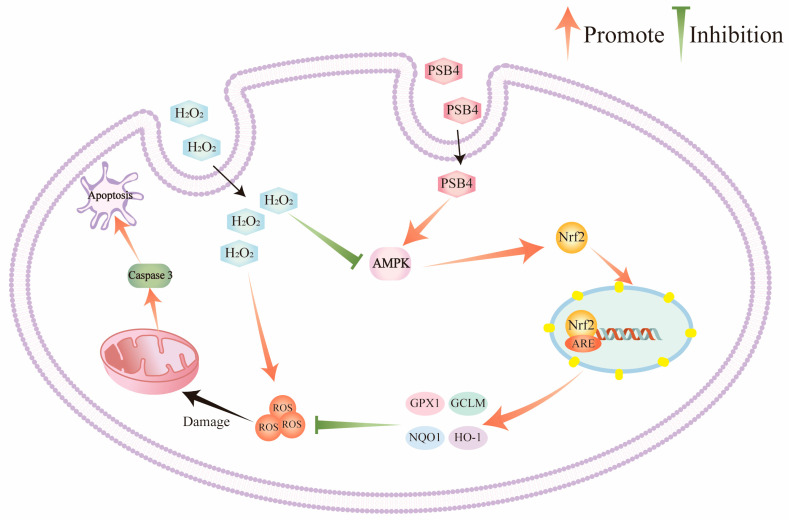
Schematic diagram of the protective mechanism of PSB4 against H_2_O_2_-induced oxidative damage in BMECs.

This study demonstrates that, at the tested concentrations, PSB4 exhibits a definite protective effect against H_2_O_2_-triggered oxidative damage in BMECs. The underlying mechanisms are as follows: PSB4 activates AMPK signaling, promotes Nrf2 nuclear translocation and upregulates antioxidant genes, thereby inhibiting excessive ROS production. Concurrently, PSB4 effectively blocks the mitochondrial apoptotic pathway by modulating the BCL2/Bax protein ratio and suppressing Caspase-3 activation. The fact that the AMPK-specific inhibitor CC antagonizes this protective effect indicates that PSB4 alleviates oxidative damage by modulating the AMPK/Nrf2/Caspase-3 signaling axis. (Figure 7).

## Figures and Tables

**Table 1:** Primer sequences used in quantitative real-time PCR.

Gene	GenBank ID	Primer Sequences (5′ to 3′)	Product Size
*GCLM*	NM_001038143.1	Forward: CAGTGGGCACAGGTAAAACC Reverse: GCTCGAATGTCAGGGATGCT	188 bp
*HO-1*	NM_001014912.1	Forward: CATCGACCCCACACCTACAC Reverse: AGACGCCATCACCAGCTTAAA	191 bp
*NQO1*	NM_001034535.1	Forward: TGGCCAATTCAGAGTGGCA Reverse: TCCAGGCGTTTCTTCCATCC	131 bp
*GPX1*	NM_174076.3	Forward: AACGTAGCATCGCTCTGAGG Reverse: GATGCCCAAACTGGTTGCAG	121 bp
*β-actin*	NM_173979.3	Forward: CACAGGCCTCTCGCCTTC Reverse: ATCATCCATGGCGAACTGGT	71 bp

## Data Availability

The original data supporting the results of this study will be provided by the authors without any undue reservation.
